# Oral 18-methoxycoronaridine activity in simian and murine *Leishmania amazonensis* infection

**DOI:** 10.3389/fphar.2026.1780255

**Published:** 2026-04-10

**Authors:** J. C. Delorenzi, T. S. Melo, F. T. Silveira, L. A. Carneiro, M. B. Campos, M. R. Cunha, E. L. Ricci, A. R. Fukushima, S. L. Hurst, S. M. Freeman, M. S. Almeida-Saavedra, A. M. Mourad, C. Sacchelli-Ramos, L. B. O. Delorenzi, G. B. Delorenzi, E. M. Saraiva

**Affiliations:** 1 Laboratory of Applied Pharmacology and Toxicology, Center of Biological and Health Sciences, Mackenzie Presbyterian University, SãoPaulo, Brazil; 2 Laboratory of Drug Development, Department of Research, Development and Innovation, Hebron Pharma Group, Recife, Brazil; 3 Evandro Chagas Institute (Secretariat for Health and Environmental Surveillance, Ministry of Health), Ananindeua, Brazil; 4 Center for Tropical Medicine, Federal University of Pará, Belém, Brazil; 5 National Primate Center, Ministry of Health, Ananindeua, PA, Brazil; 6 Department of Organic Chemistry and Center of Medicinal Chemistry (CQMED), Center for Molecular Biology and Genetic Engineering (CBMEG), University of Campinas, UNICAMP, SP, Brazil; 7 Department of Pathology, Faculty of Veterinary Medicine and Animal Sciences, University of São Paulo, SãoPaulo, Brazil; 8 Universidade Santo Amaro (UNISA), SãoPaulo, Brazil; 9 MindMedicine, InC, New York, NY, United States; 10 Laboratory of Innate Immunity, Department of Immunology, Paulo de Góes Microbiology Institute, Universidade Federal do Rio de Janeiro (UFRJ), Rio deJaneiro, Brazil

**Keywords:** 18-methoxycoronaridine, chlorocebus aethiops, leishmania amazonensis, *Macaca fascicularis*, murine experimental models, oral treatment, simian experimental models, cutaneous leishmaniasis

## Abstract

**Introduction:**

Leishmaniasis remains a major unmet medical need, with limited oral options and persistent constraints related to efficacy, tolerability, and treatment duration. We investigated 18-methoxycoronaridine (18-MC), an iboga-type indole alkaloid, as an oral candidate against *Leishmania amazonensis,* integrating rodent and non-human primate efficacy, toxicology, and pharmacokinetics to support clinical translation.

**Methods:**

Antileishmanial activity was first assessed in BALB/c mice treated orally with 18-MC (20 mg/kg/day). Translational efficacy was evaluated in non-human primates receiving oral 18-MC at 30 or 90 mg/kg/day for 28 days. Safety was characterized through acute and repeated-dose toxicology to establish the no-observed-adverse-effect level (NOAEL), complemented by pharmacokinetic analyses.

**Results:**

A 5-day oral regimen of 18-MC reduced murine tissue parasite burden (expressed as viable parasites per whole organ) by >99%. In* Chlorocebus aethiops*, 18-MC produced an exposure-dependent reduction in lesion burden, achieving ∼98% inhibition at 90 mg/kg/day, with marked re-epithelialization and no persistent clinical abnormalities. Pharmacokinetic modeling demonstrated a steep exposure–response relationship (EC50 ≈ 209 ng⋅h/mL) and a therapeutic index of approximately 5.4. The NOAEL was 50 mg/kg/day across species.

**Discussion:**

18-MC demonstrates potent orally bioavailable antileishmanial activity with rodent–primate translational concordance and a safety profile compatible with first-in-human evaluation, supporting Phase I advancement with an estimated human equivalent dose of ∼16 mg/kg/day.

## 1 Introduction

Leishmaniasis remains a major global health priority, with more than one billion people living in endemic areas at risk of infection and an estimated 600,000–1,000,000 new cases of cutaneous leishmaniasis (CL) occurring annually worldwide (World Health Organization, 2023; Sunyoto et al., 2018). In the Americas—including Brazil—multiple clinical phenotypes are observed, ranging from localized cutaneous disease to mucosal involvement and, less commonly, disseminated and anergic diffuse presentations, with *Leishmania (L.) amazonensis* often implicated in severe immunopathological spectra (Silveira et al., 2009; Silveira, 2019; Silveira, 1972; Sghaier et al., 2022). Despite decades of clinical experience, therapeutic progress has been limited, and outcomes remain heterogeneous across parasite species, regions, and host factors: for example, cure rates reported for systemic pentavalent antimonials in the Americas span from less than 50% in some settings to more than 90% in others (Castro et al., 2023). Miltefosine—an alkylphosphocholine and the only orally administered drug currently registered for leishmaniasis—has improved programmatic feasibility, yet clinical effectiveness is similarly variable, with New World CL cure rates differing markedly by species and geography (e.g., ∼33%–82% across species in clinical datasets) (Sunyoto et al., 2018; Machado et al., 2010; CDC, 2024). Together, these features underscore the need for safe, scalable, and more consistently effective oral regimens that can shorten treatment courses and improve outcomes across diverse epidemiological contexts (World Health Organization, 2023).

18-Methoxycoronaridine (18-MC) is a synthetic iboga-type indole alkaloid congener developed from the ibogaine scaffold and initially advanced in neuropharmacology as a candidate for substance use disorders, aiming to retain anti-addictive activity while mitigating key liabilities described for ibogaine (Glick et al., 1996; Glick et al., 2000; Maisonneuve and Glick, 2003). Glick and his team demonstrated that 18-MC reduced self-administration of drugs of abuse and attenuated withdrawal-related behaviors, with convergent mechanistic evidence implicating modulation of nicotinic acetylcholine receptor signaling—particularly via antagonism of α3β4-containing receptors within discrete circuits linked to reinforcement (Pace et al., 2004; Glick et al., 2011). Beyond addiction models, 18-MC has also emerged as an anti-infective scaffold: early work demonstrated *in vitro* activity of 18-MC (and related iboga alkaloid congeners) against intracellular amastigotes of *L. amazonensis*, supporting its repositioning potential for leishmaniasis drug discovery (Delorenzi et al., 2002). Given that rodent efficacy alone is frequently insufficient to predict clinical performance, non-human primate (NHP) models provide a strategically important translational bridge due to closer phylogenetic, physiological, and immunological proximity to humans; NHP models have been specifically discussed as valuable platforms to de-risk antileishmanial development and to strengthen translational inference (Olobo et al., 2001; Harding, 2017; André et al., 2020). Accordingly, this study aimed to evaluate the oral leishmanicidal activity of 18-MC in murine and simian models of *L. amazonensis* infection and to characterize its safety profile support clinical translation.

## 2 Materials and methods

### 2.1 Ethics approval

The mice experimental procedures adopted for this study followed the standards established by the Research Ethics Committee of Mackenzie Presbyterian University (CEUA 046/2009). Non-human primates (NHP) studies followed the standards of Research Ethics Committee of Evandro Chagas Institute–IEC (CEUA 41/2015).

### 2.2 Parasites

For mouse experiments, promastigotes of *Leishmania* (*L*.) *amazonensis* (strain WHOM/BR/75/JOSEFA) were cultured at 26 °C in medium 199 (Cultilab, São Paulo, Brazil) supplemented with 10% fetal bovine serum (Cultilab), 2% human urine, and 50 μg/mL gentamicin (Mantecorp, Rio de Janeiro, Brazil), hereinafter referred to as Culture Medium. For the NHP assays, promastigotes of *L*. (*L*.) *amazonensis* (strain IFLA/BR/2015/Bragança), the prevalent strain in Pará State, Brazil, were used. This strain was freshly isolated from the phlebotomine vector *Lutzomyia flaviscutellata* and cultured under the same conditions.

### 2.3 Animals

Male and female BALB/c mice, approximately 4 weeks old and weighing 20–25 g, were purchased from the Central Animal Facility of the Federal University of São Paulo (UNIFESP). Animals were housed under controlled conditions (22 °C–25 °C, 12 h light/dark cycle) with access to food and water *ad libitum*. Their weight was monitored daily during the experimental period.

Males and females *Chlorocebus aethiops* monkeys (1.5–3 years old) from the Primates National Center (CNP/SVS/MS, Ananindeua, Pará, Brazil) were maintained in standard-sized, isolated cages in an isolated primate vivarium at CNP-IEC with specific diet conditions, composed by milk, eggs, a variety of fruits and vegetables, balanced commercial feed, and water *ad libitum*.

Male and female experimentally naïve Cynomolgus monkeys (*Macaca fasciculata*), approximately 2 years and 4 months to 3 years and 7 months of age at transfer, were maintained at the same conditions already described for monkeys. These animals were originally received from Covance Research Products, Inc. The country of origin for all monkeys was Vietnam. Animals were euthanized humanely with ketamine/xylazine overdose (90/10 mg/kg).

### 2.4 18-Methoxycoronaridine (18-MC)

**FIGURE 1:**
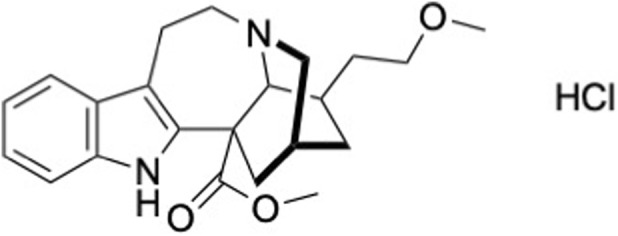
Chemical structure of 18-MC hydrochloride (C_22_H_29_ClN_2_O_3_, MW 404.93 g/mol).

The water-soluble alkaloid 18-methoxycoronaridine hydrochloride (18-MC, 98.4% purity) (Figure 1) was kindly provided by Savant HWP (now Mind Medicine) and synthesized according to Kuehne et al. (2003) by Aesica Pharmaceuticals Ltd, now Recipharm (Cheadle, United Kingdom). The schematic description of manufacturing process is shown in Supplementary Materials.

### 2.5 Leishmanicidal activity

#### 2.5.1 Murine

BALB/c mice were infected in the right footpad with 1.10^7^ stationary-phase *L. amazonensis* promastigotes. After 30 days of infection, the animals were randomly assigned to study groups. Groups of five mice received an oral dose of 18-MC (20 mg/kg) in distilled aqueous solution, once a day, for either 5 or 10 consecutive days. Glucantime (200 mg/kg, intraperitoneal) served as the positive control, while the negative control received only filtered water orally. Three days after the end of treatment, the animals were euthanized. The entire paw, cut at the ankle, and the regional lymph node (popliteal) were excised to assess the presence of parasites in the tissues.

Parasite detection was performed using the limiting dilution method described by Lima et al. (1997). Briefly, infected tissue was cut into small pieces, homogenized, and placed in 1 mL of culture medium 199 supplemented with 10% fetal bovine serum, 2% human urine, and 150 μg/mL gentamicin. The homogenates were transferred to 96-well flat-bottomed plates, with 24 dilutions performed per titer.

Parasite burden was estimated based on promastigote growth at the highest dilution after 7 days at 26 °C and is expressed as the estimated number of viable parasites per whole organ (paw or popliteal lymph node).

#### 2.5.2 Non-human primates

12 young specimens of *Chlorocebus aethiops* monkeys (7 male and 5 female) were intradermal inoculated with 10^6^ cultured stationary-phase promastigotes of *L. amazonensis*, in five different points on tail dorsal surface. Monkeys were randomized into three groups (n = 4 each) 1 month post-infection: untreated control (5% dextrose aqueous solution), 18-MC 30 mg/kg/day, once a day, for 28 days, or 18-MC 90 mg/kg/day, once a day, for 28 days 18-MC were orally administrated and lesions were bidimensional pachymetry analyzed weekly. Dose selection was based on translational scaling from murine efficacy studies (10–20 mg/kg/day) and on the need to characterize exposure–response relationships in a higher species (better explained in Section 2.10).

### 2.6 Mice acute toxicity

Mice were randomly divided into five groups (n = 5 animals/group), deprived of food for 4 h before and 2 h after treatment with 50, 100, 200 or 300 mg/kg of 18-MC distilled aqueous solution, while maintaining access to water. After administration, animals were closely observed for 2 h, on a flat, enclosed surface. After the first 2 h period, observations were made at regular 4 h intervals on the first day and every 24 h for 14 days. Behavioral parameters were monitored, including stereotyped movements, locomotion, depression, seizures, sedation, tremors, piloerection, tail erection, and lethality, following Roux et al. (2005). After 14 days animals were euthanized for necropsy and tissue (liver, heart, brain kidney and blood) were collected for histopathological and hematological analysis.

### 2.7 Mice repeated-dose toxicity

For repeated-dose toxicity, BALB/c mice approximately 6 weeks old and weighing 25 g were randomly divided into 4 groups and treated orally with 18-MC at 2, 10, 50 mg/kg in distilled water once daily for 30 days. The control group (which received only water) and the group treated with 50 mg/kg comprised 20 animals, whereas the groups treated with 2 and 10 mg/kg comprised 10 animals each. Male and female BALB/c mice from the same litter were used for each experiment.

Toxicity measurements were done as described for acute toxicity. After 30 days of observation (counting from the last drug administration day), animals were euthanized for necropsy, hematological and clinical chemistry analysis.

### 2.8 Murine LD_50_

Twenty-four hours after drug administration in acute toxicity assessment, the lethal dose (LD_50_) was estimated using linear regression according to the methodology proposed by Miller and Tainter in 1944 (Randhawa, 2009).

### 2.9 Non-human primates toxicity

Toxicity testing in non-human primates was conducted at the MPI Institute, in partnership with Savant HWP (now Mind Medicine) and our group. A total of 22 male and 22 female experimentally naïve Cynomolgus monkeys were transferred from the stock colony, as described above.

Using a standard randomization procedure based on weight and measured value, 20 male and 20 female animals (weighing between 2.36 and 3.11 kg and between 2.22 and 3.26 kg, respectively, at the time of randomization) were allocated to the control and treatment groups. Vehicle (5% dextrose aqueous solution) and 18-MC were administered once daily for 14 consecutive days during the study via oral gavage. The monkeys were fasted for at least 2 h prior to the first animal dosed and for at least 2 h following the last animal dosed. The dose levels were 50, 150, and 400/300 mg/kg/day. A detailed clinical examination of each animal was performed pretest (Day −1) and on Days 1, 3, 6, 9, 12, and 15 (1–2 h postdose) during the study and twice week (Days 18, 24, 27, 30, 33, 36, 39, and 42) during the recovery period. On occasion, clinical observations were recorded at unscheduled intervals. The observations included, but were not limited to, evaluation of skin, fur, eyes, ears, nose, oral cavity, thorax, abdomen, external genitalia, limbs and feet, respiratory and circulatory effects, autonomic effects such as salivation, and nervous system effects including tremors, convulsions, reactivity to handling, and unusual behavior. Bone marrow smears from all animals were evaluated microscopically. Complete differentials and myeloid: erythroid (ME) ratios were performed. Blood samples (approximately 1 mL) were collected from all animals via the femoral artery/vein for drug plasma concentrations determination. Samples were collected pre-dose and at 1, 4-, 8-, 12-, and 24 h post-dose on Days 1 and 14. The animals were not fasted prior to blood collection, except for the intervals that coincided with fasting for clinical pathology collections.

### 2.10 Pharmacokinects modeling

Pharmacokinetic simulations were performed using deterministic computational scripts implemented in Python (version 3.x). Numerical calculations were conducted using NumPy, and graphical visualization was prepared using GraphPad Prism 10.

A one-compartment model with first-order absorption and elimination was assumed. Systemic exposure (AUC) was estimated assuming linear pharmacokinetics within the evaluated dose range, based on previously established toxicokinetic data in monkeys (Rowland and Tozer, 2011; Overgaard et al., 2015; Gabrielsson, 2016):

$${AUC}_{dose}={AUC}_{reference}\times \frac{Dose}{Reference\;Dose}$$

where the reference AUC was 333 ng·h/mL at 50 mg/kg/day.

Leishmanicidal efficacy was modeled using a classical E_max_ model (Pereira et al., 2022):

$$E=\frac{E_{\max}\cdot AUC}{EC_{50}+AUC}$$

Parameter estimation (E_max_ and EC_50_) was performed via grid-search optimization minimizing squared prediction error (Zou et al., 2020).

The hematologic toxicity threshold was previously modeled using an analogous E_max_ framework, with an estimated EC_50_ of 1,136 ng·h/mL.

The therapeutic index was defined as:

$$TI=\frac{EC50_{toxicity}}{EC50_{efficacy}}$$

### 2.11 Statistics

All results were expressed as mean ± standard error of the mean (SEM). When variance was homogeneous, groups were compared using analysis of variance (ANOVA), followed by the Newman-Keuls or Tukey tests. In cases of inhomogeneous variance, analyses followed nonparametric methods such as the Kruskal–Wallis test, followed by Dunn’s test, when necessary. was considered significant. Statistical analyses were performed using a computer program and GraphPad Prism 10 PRO® software. Significance was defined as p < 0.05 (*), and p < 0.01 (**).

## 3 Results

### 3.1 Murine anti-leishmanial activity

18-MC leishmanicidal activity was determined in a model of established (chronic) infection (Le Fichoux et al., 1998). Each mouse was infected with 1.10^7^ *L. amazonensis* promastigotes in stationary phase, and treatment began on day 30 (D30) post-infection. Oral administration of 18-MC (20 mg/kg/day) for 5 or 10 consecutive days produced marked reductions in parasite burden, expressed as viable parasites per whole organ. In infected paws, 18-MC reduced parasite load by 99.9% after 5 days and 99.98% after 10 days of treatment when compared with the untreated control. Glucantime (200 mg/kg/day, IP) achieved reductions of 99.0% and 99.4%, respectively, when compared with the untreated control. In popliteal lymph nodes, parasite reduction reached 99.9% for 18-MC and 99.8% for Glucantime after 5 days when compared with the untreated control (Table 1).

**TABLE 1:** Leishmanicidal activity of 18-MC in the murine model.

Parasitary load				
Drug [mg/kg/day]	Paw		Lymph node	
​	Parasites (x 10^3^)	% reduction	Parasites (x 10^3^)	% reduction
CTRL	54,6	0	65,5	0
GLU 200 IP	0,55*	99	0,13*	99,8
18-MC 20 VO	0,15*	99,7	0,12*	99,9

BALB/c mice were infected with *L. amazonensis* promastigotes (10^6^) in the right posterior plantar pad. Treatment was initiated on the 30th day after infection (D30) with five consecutive treatments. Results from three independent experiments are expressed as number of viable parasites per whole organ, determined by limiting dilution assay and as the percentage of parasite load reduction compared to the untreated control. CTRL, untreated control; GLU, 200 IP, Glucantime intraperitoneal. 18-MC VO, 18-methoxycoronaridine via Oral. *p < 0.001.

### 3.2 Non-human primates anti-leishmanial activity

**FIGURE 2:**
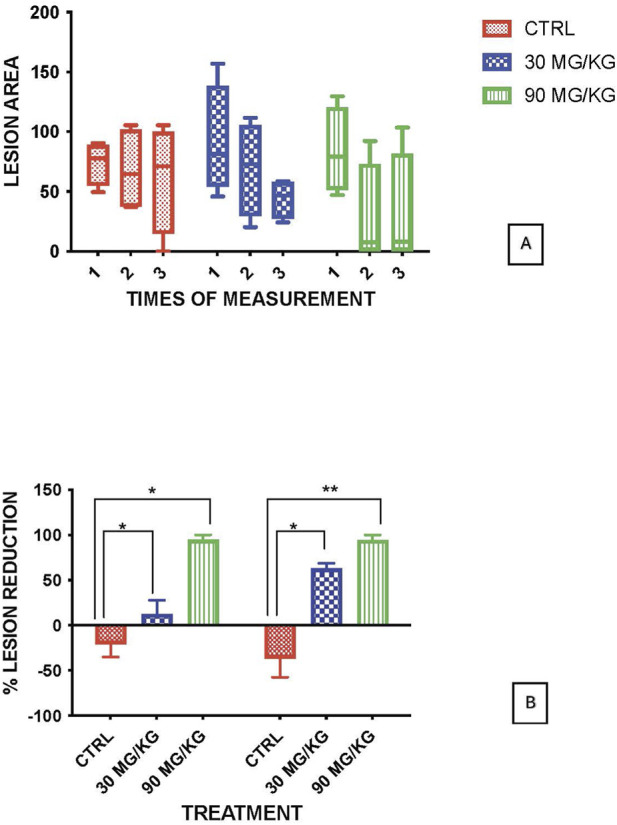
Anti-leishmanial activity of 18-MC in non-human primates. **(A)** Lesion área (mm^2^) measured at: (1) baseline (pre-dose, Day 0), (2) Day 14 of treatment, and (3) Day 30 of treatment. **(B)** Percentage reduction in lesion area, comparing baseline with Day 14 and Day 30 measurements.

**FIGURE 3:**
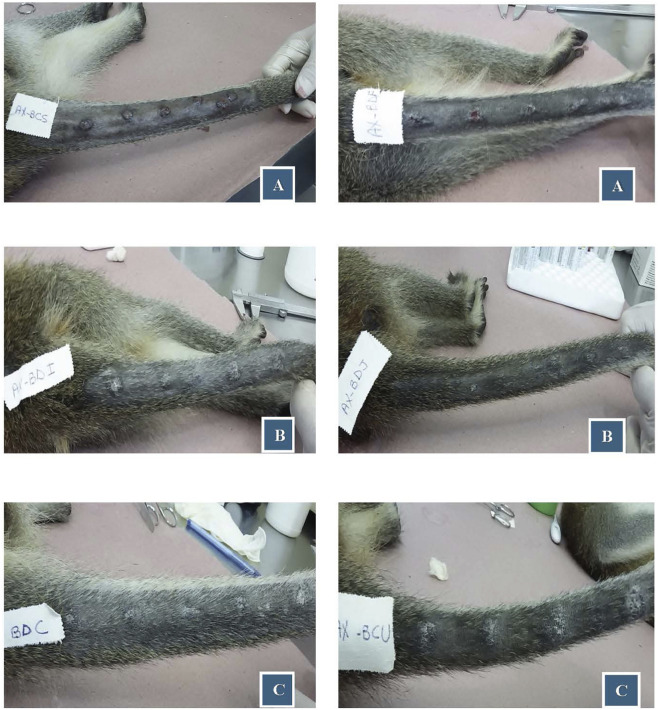
Antileishmanial activity of 18-MC in non-human primates. Lesion characteristics on Day 60 post-infection: **(A)** Control (active, 65%; active transitioning to healing, 5%; satellite lesions, 0%; healed, 30%); **(B)** 30 mg/kg/day (active, 0%; active transitioning to healing, 10%; satellite lesions, 10%; healed, 80%); **(C)** 90 mg/kg/day(active, 0%; active transitioning to healing, 0%; satellite lesions, 0%; late-stage lesion regression, 20%; healed, 80%).

**FIGURE 4:**
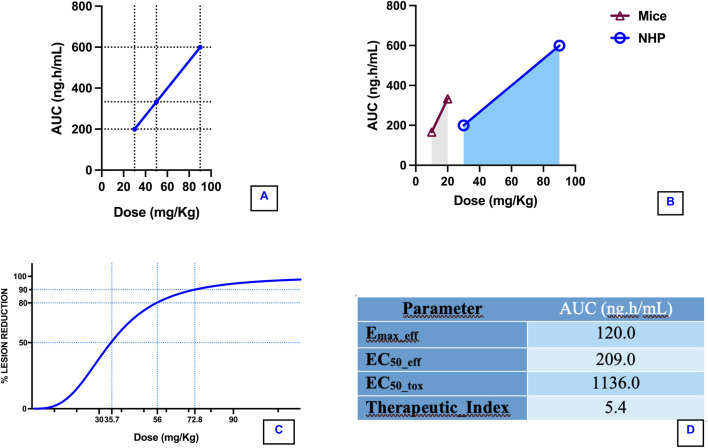
Integrated pharmacokinetic–pharmacodynamic and toxicokinetic modeling of 18-MC in non-human primates. **(A)** Dose–exposure relationship demonstrating near-linear pharmacokinetics across the evaluated range (30–90 mg/kg), with systemic exposure expressed as area under the plasma concentration–time curve (AUC, ng·h/mL). The proportional increase in AUC supports dose-dependent systemic availability. **(B)** Cross-species exposure comparison between murine and non-human primate (NHP) models, highlighting differences in systemic exposure at comparable dose levels and supporting translational extrapolation. **(C)** Sigmoidal dose–response curve describing percentage lesion reduction as a function of administered dose (mg/kg), modeled using a four-parameter logistic function (Bottom fixed at 0%, Top fixed at 100%). The estimated pharmacodynamic parameters were: EC_50_ = 35.3 mg/kg (AUC ≈235 ng·h/mL); EC_80_ = 55.7 mg/kg (AUC ≈371 ng·h/mL); EC_90_ = 72.8 mg/kg (AUC ≈485 ng·h/mL); Hill coefficient = 3.04, indicating a steep exposure–efficacy relationship. **(D)** Integration of pharmacodynamic and toxicokinetic parameters. EC50_eff (AUC = 209 ng·h/mL) represents the exposure associated with 50% of maximal therapeutic effect. EC50_tox (AUC = 1,136 ng·h/mL) represents the exposure associated with 50% of toxicological response, derived from toxicokinetic modeling. The calculated Therapeutic Index (TI = 5.4) reflects the separation between efficacious and toxic exposures.

Animals were intradermal inoculated with 10^6^ cultured stationary-phase promastigotes of *L*. *amazonensis*, in five different points on tail dorsal surface. After 30 days of infection, they were treated with 18-MC (30 mg/kg/day and 90 mg/kg/day) for 28 days. Treatment produced dose-dependent reduction in lesion area, with decreases of 67% at 30 mg/kg and 98% at 90 mg/kg by 2 months post-infection. Moreover, when compared to controls lesions, treated animals exhibited better re-epithelization and higher number of cured lesions per animals with no or less satellite lesions (Figures 2, 3). Exposure–response analysis demonstrated a clear Emax-type relationship between systemic exposure and leishmanicidal efficacy. The estimated EC_50_ for efficacy was 209 ng·h/mL, while the previously modeled EC_50_ for hematologic toxicity was 1,136 ng·h/mL. This separation between efficacious and toxicity-associated exposure levels suggests a favorable therapeutic margin in non-human primates (Figure 4).

### 3.3 Murine acute toxicity

The protocol used for acute toxicity is the classic one proposed by the Organization for Economic Co-operation and Development (OECD) and recommended by the Brazilian Health Regulatory Agency (ANVISA), using male and female BALB/c mice from the same litter in each experiment. Animals were treated with 50, 100, 200 and 300 mg/kg of 18-MC. In males, treatment with 50 mg/kg of 18-MC induced drowsiness and prostration, recovering along the 14 days. At 100 mg/kg, death has been observed in males, along with seizures, tremors, and respiratory difficulties. At doses of 200 and 300 mg/kg of 18-MC, 100% of male mice and 43% of female mice induced death. The estimated LD_50_ for 18-MC was approximately 100 mg/kg in males and 200 mg/kg in females (Table 2).

**TABLE 2:** Murine acute toxicity of 18-MC. 18-MC was administered as a single dose at concentrations of 50, 100, 200, and 300 mg/kg, orally (PO). **♂** = males and **♀** = females. Control group animals received drinking water via the same route of administration.

Treatment	Mortality	Symptoms description
CTRL	0	No symptoms or deaths
18-MC 50 mg/kg (n = 5 ♀ and 8 ♂)	0	**♀** – No symptoms or deaths **♂** – Animals were drowsy during the first 15 min following treatment. After 1 hour, all animals were responsive to stimuli. Fighting decreased significantly
18-MC 100 mg/kg (n = 3 ♀ and 13 ♂)	0 **♀** 3 **♂**	**♀** - **1**st hour: Animals were lethargic; two developed diarrheas; increased water intake. After 2 h, the animals recovered; after 4 h, they resumed normal feeding. After 7 days: Animals showed normal activity; water and food intake were unchanged ♂ - 1st hour: *Tremor:* Animals exhibited tremors 30 min after oral administration. They returned to normal after 180 min. *Motor incoordination/balance*: Animals showed difficulty ambulating 30 min after gavage. They returned to normal after 180 min. *Other signs*: Labored/irregular breathing, drowsiness, lethargy, vasodilation (increased vascularization), lacrimation, and defecation. These signs resolved within 180 min after treatment. *Death*: One animal died 180 min after treatment, and two others died after 24 h. Signs observed prior to death were tremors, prostration, and seizures. Necropsy did not reveal any organic lesions that would explain the seizures
18-MC 200 mg/kg (n = 7 ♀ and 4 ♂)	3 **♀** 4 **♂**	**♀** - 1st hour: Animals were prostrated; two developed diarrheas; water intake increased. One animal was cachectic. From the 2nd hour onward, 1 animal developed a seizure and progressed to death 2.5 h after treatment. The second and third animals were lethargic and unresponsive to stimuli. The remaining animals were normal. After 2 days: The second and third animals died, presenting evidence of internal hemorrhage. After 7 days, the surviving animals were clinically normal ♂ - animals exhibited seizures, tremors, and motor incoordination within the first hours after treatment. All animals died within 180 min. Clinical signs observed prior to death included tremors, prostration, and seizures. Necropsy did not reveal any organic lesions that would account for the seizures
18-MC 300 mg/kg (n = 7 ♀ and 4 ♂)	3 ♀ 4 ♂	♀ - 1st hour: Animals were prostrated; 4 developed diarrheas; water intake increased. Two animals were cachectic. From the 2nd h onward: Two animals developed seizures and died 2.5 and 3.25 h after treatment, respectively. The third animal remained lethargic and unresponsive to stimuli. The remaining animals were normal. After 5 days: The third animal died following a seizure episode. After 7 days: The surviving animals were clinically normal ♂ - animals exhibited seizures, tremors, and motor incoordination within the first hours after treatment. All animals died within 180 min. Clinical signs observed prior to death included tremors, prostration, and seizures. Necropsy did not reveal any organic lesions that would account for the seizures

### 3.4 Murine repeated-dose toxicity

After daily oral administration of 18 MC, the animals were monitored for 180 min. During this period, no signs of toxicity were observed. The protocol employed follows the classical guidelines proposed by the OECD (2001) and recommended by ANVISA (2013). The animals exhibited none of the signs of toxicity observed in acute toxicity studies (highest doses).

Animals in the 50 mg/kg group showed some signs of toxicity, particularly among females, where five deaths occurred. These deaths were attributed to animals’ low body weight at randomization (less than 20 g), resulting in doses exceeding 75 mg/kg rather than the intended 50 mg/kg. Additionally, some animals displayed signs of weakness prior to treatment initiation. Animals with appropriate body weights prior to the treatment initiation did not display any signs of toxicity (Table 3).

**TABLE 3:** Murine repeated dose toxicity of 18-MC. 18-MC was administered orally at doses of 2, 10, and 50 mg/kg ♂ = males and ♀ = females. Control group animals received drinking water via the same route of administration.

Treatment	Mortality	Symptoms description
CTRL (n = 20 ♀ and 19 ♂)	0 ♀ 1 ♂	Death: The animal had shown signs of weakness since the beginning of the protocol due to low weight at the time of randomization
18-MC 2 mg/Kg (n = 10 ♀ and 10 ♂)	0 ♀ 0 ♂	♀ – The animals did not exhibit any physiological or behavioral changes ♂ – The animals did not exhibit any physiological or behavioral changes
18-MC 10 mg/Kg (n = 10 ♀ and 10 ♂)	0 ♀ 0 ♂	♀ – The animals did not exhibit any physiological or behavioral changes ♂ – One animal developed a lesion in the vibrissae (whisker) region, probably not related to the treatment
18-MC 50 mg/Kg (n = 20 ♀ and 20 ♂)	5 ♀^a^ 0 ♂	♀ – 1st hour: One animal died shortly after treatment. After 24 h: Another animal died; the cause of death could not be determined. Three animals were severely debilitated, unable to move their hind limbs, and exhibited tremors, but responded to touch. They were weighed and found to be < 20 g; therefore, they were receiving a dose >50 mg/kg. These animals were removed from the treatment. After 2 days: One animal was found dead. After 7 days: The surviving animals were clinically normal ♂ – After 13 days: Three animals developed diarrhea. After 17 days: One animal presented blood-stained mucus in the anal region and diarrhea

^a^ In the 50 mg/kg group, toxicity signs and five deaths (mainly in females) were observed; however, these outcomes were attributed to low body weight at randomization (<20 g), which resulted in an effective dose >75 mg/kg, and to pre-existing weakness in some animals. In contrast, animals with appropriate body weight and normal baseline clinical status showed no evidence of toxicity.

Blood samples collected at euthanasia were analyzed for hematological and biochemical parameters as detailed in the Materials and Methods section. The results indicate that treatment with 18-MC did not induce any hematological changes in female mice. All parameters remained within normal reference ranges. Although slight variations in neutrophil and lymphocyte counts were observed in the control group, these were not statistically significant (Tables 4 and 5). Biochemical parameter analyses (glucose, cholesterol, and triglycerides) in female and male mice treated with 18-MC for 30 days. No statistically significant biochemical changes were observed between the treated and untreated control groups in either male or female mice, indicating the absence of biochemical toxicity markers (Tables 4 and 5).

**TABLE 4:** Hematological and biochemical parameters of female mice treated with different doses of 18-MC for 30 days (P > 0.05).

Treatment	Hematocrit (%)	Neutrophils (%)	Lymphocytes (%)	Monocytes (%)	Basophils (%)	Eosinophils (%)	Glucose (Mean ± SE)	Cholesterol (Mean ± SE)	Triglycerides (Mean ± SE)
Control	51.5	45	55	0	0	0	229.6 ± 54.1	44.4 ± 1.8	37.7 ± 2.1
2 mg/kg	52.1	21	79	0	0	0	78.7 ± 0.01	47.0 ± 1.8	28.2 ± 1.2
10 mg/kg	46.9	24	76	0	0	0	178.4 ± 14.9	50.3 ± 1.1	51.6 ± 9.8
50 mg/kg	50.9	23	77	0	0	0	200.4 ± 13.9	45.7 ± 2.5	49.2 ± 4.5
Reference values^a^	43.2–56.3	20–30	70–80	0–2	0–2	0–7	115.01 ± 11.34	61.03 ± 17.37	101.33 ± 10.84

^a^ Reference biochemical values from Spinelli et al. (2014).

**TABLE 5:** Hematological and biochemical parameters of male mice treated with different doses of 18-MC for 30 days (P > 0.05).

Group	Hematocrit (%)	Neutrophils (%)	Lymphocytes (%)	Monocytes (%)	Basophils (%)	Eosinophils (%)	Glucose (Mean ± SE)	Cholesterol (Mean ± SE)	Triglycerides (Mean ± SE)
Control	45.4	22	74	3	0	1	149.9 ± 50.3	51.6 ± 2.8	41.2 ± 1.7
2 mg/kg	45.0	30	57	10	0	3	213.8 ± 23.2	48.0 ± 3.2	34.1 ± 2.2
10 mg/kg	46.0	32	62	6	0	0	162.3 ± 78.6	59.1 ± 2.1	49.7 ± 5.3
50 mg/kg	45.8	35	61	3	0	1	124.2 ± 18.9	44.4 ± 1.5	45.5 ± 3.4
Reference values^a^	42.7–52.9	20–30	70–80	0–2	0–2	0–7	115.01 ± 11.34	61.03 ± 17.37	101.33 ± 10.84

^a^ Reference biochemical values (as provided) Spinelli et al. (2014).

Normal reference values for biochemical parameters in mice vary in the literature. The reference values from Spinelli et al. (2014) were used because they were derived from animals housed under the same conditions as those in our studies.

Based on clinical symptomatology, hematological, biochemical, and histopathological analyses following 30 consecutive days of treatment in male and female mice, it can be concluded that the 18-MC exhibits low toxicity. The Non-Observed Adverse Effect Level (NOAEL) for repeated-dose administration of 18-MC over 30 days is established at 50 mg/kg for both male and female mice.

### 3.5 Non-human primates repeated dose toxicity

18-MC-related clinical signs noted at 400 mg/kg/day (Day 1) included eye lid(s) partially or completely closed, decreased activity, hunched posture, and salivation all with a low number of animals affected and low incidence. At 300 mg/kg/day, 18-MC-related clinical signs of decreased activity, ataxia, inappetence, salivation, hunched posture, emesis, vomitus, and eyelid partially/completely closed were noted, all with low incidence. At 150 mg/kg/day 18-MC-related signs of decreased activity, inappetence, eyelid partially/completely closed were noted all with a low number of animals affected and low incidence. These changes had resolved by the end of the recovery interval, except for inappetence in one female at 150 mg/kg/day. Additional signs noted at ≥ 50 mg/kg/day that were related to the administration of 18-MC were watery and/or soft feces, vomitus and/or emesis, all with a low number of animals affected and low incidence. These changes had resolved by the end of the recovery interval. 18-MC-related findings seen during functional observational battery evaluations were limited to that which had already been seen during the detailed clinical examinations: hunched posture, eyes slightly drooping, and slight decreases in body temperature. These changes had resolved by the recovery interval.

Repeated oral administration of 18-MC for 14 days induced dose-dependent hematologic modulation. In males, hemoglobin decreased up to 20% and total leukocytes decreased 40% at ≥150 mg/kg. In females, leukocyte reductions were observed at 50 mg/kg.

Clinical chemistry showed mild decreases in alkaline phosphatase and total bilirubin without consistent ALT/AST elevations. No structural liver pathology was reported. All findings were fully reversible during the recovery period.

Following 14 days of oral 18-MC administration to male and female monkeys the NOAEL was 50 mg/kg/day, considering a very conservative approach for this determination (Table 6).

**TABLE 6:** Summary of key detailed clinical observations NHP toxicity (Days 1–14). Values are number of animals affected (M/F). “0/0” = not observed. “NR” = not reported in the incidence summary table.

Clinical sign	0 mg/kg/day	50 mg/kg/day	150 mg/kg/day	300 mg/kg/day	400→300 mg/kg/day†
Eyelid(s) partially/completely closed	0/0	0/0	1/1	0/1	1/1
Decreased activity	0/0	0/0	2/0	3/3	2/0
Hunched posture	0/0	0/0	2/1	3/2	1/0
Salivation	0/0	0/0	1/0	2/0	1/0
Ataxia	0/0	0/0	0/0	0/1	0/0
Inappetence	0/0	0/0	1/2	4/2	0/0
Emesis (any)^‡^	1/1	1/1	1/2	1/1	0/0
Vomitus (any)^‡^	0/0	0/0	2/1	2/2	0/0
Feces soft	0/1	3/0	3/0	3/2	2/3
Feces watery	0/0	4/0	3/3	2/1	NR

^†^High-dose group received 400 mg/kg/day on Day 1, then 300 mg/kg/day on Days 2–7 (dose-volume adjustment).

^‡^“Any” = maximum animals affected across descriptors reported within the same dose/sex line (conservative union).

Recovery: signs resolved by end of recovery except inappetence in one female at 150 mg/kg/day.

**TABLE 7:** Hematological parameters in male *Cynomolgus monkeys* after 14-day oral administration of 18-MC.

Parameter	Control	50 mg/kg	150 mg/kg	400/300 mg/kg	Significance
Hemoglobin	—	NC	−17%*	−20%**	Dose-dependent decrease
Total leukocytes	—	NC	NC	−40%**	Significant at high dose
Neutrophils	—	NC	NC	−20%*	Mild decrease
Lymphocytes	—	NC	NC	−45%**	Marked decrease
Monocytes	—	NC	NC	−84%**	Marked decrease

Data represent percent change vs. pretest mean.

NC, no clinically relevant change.

*p < 0.05 vs. control (Dunnett’s test).

**p < 0.01 vs. control (Dunnett’s test).

All findings were fully reversible during recovery.

**TABLE 8:** Hematological parameters in female cynomolgus monkeys after 14-day oral administration of 18-MC.

Parameter	Control	50 mg/kg	150 mg/kg	400/300 mg/kg	Significance
Hemoglobin	—	NC	−19%*	−17%*	Moderate decrease
Total leukocytes	—	−29%*	−33%**	−45%**	Dose-dependent
Neutrophils	—	−29%*	−36%**	−47%**	Dose-dependent
Lymphocytes	—	−26%*	−28%*	−41%**	Dose-dependent
Monocytes	—	NC	−81%**	−77%**	Marked decrease

All findings were reversible.

Significance determined using ANOVA, followed by Dunnett’s *post hoc* test.

**TABLE 9:** Clinical chemistry findings in male cynomolgus monkeys.

Parameter	50 mg/kg	150 mg/kg	400/300 mg/kg	Interpretation
ALT	NC	NC	Isolated ↑ (1 animal)	Not dose-consistent
AST	NC	NC	Isolated ↑ (1 animal)	Not dose-consistent
Alkaline phosphatase	Mild ↓*	Mild ↓*	Mild ↓*	Reversible
Total bilirubin	Mild ↓*	Mild ↓*	Mild ↓*	Reversible
Creatinine	NC	Isolated ↑ (1 animal)	NC	Sporadic

No dose-dependent hepatocellular injury pattern observed.

**TABLE 10:** Clinical chemistry findings in female cynomolgus monkeys.

Parameter	50 mg/kg	150 mg/kg	400/300 mg/kg	Interpretation
ALT	NC	NC	NC	No hepatotoxicity
AST	NC	NC	NC	No hepatotoxicity
Alkaline phosphatase	Mild ↓*	Mild ↓*	Mild ↓*	Reversible
Total bilirubin	Mild ↓*	Mild ↓*	Mild ↓*	Reversible

*p < 0.05 vs. control.

### 3.6 Pharmacokinetic modeling

Exposure–response relationships were characterized using a standard E_max_ model, as originally described by Holford and Sheiner (1981), and translational exposure comparisons across species were interpreted using established interspecies scaling principles (Mahmood et al., 2003; Reagan-Shaw et al., 2008).

Systemic exposure in *C. monkeys* was estimated from previously characterized toxicokinetic data, which demonstrated linear pharmacokinetics within the evaluated dose range. With an exposure reference of 333 ng·h/mL at 50 mg/kg/day, the estimated AUC values were 200 ng·h/mL at 30 mg/kg/day and 600 ng·h/mL at 90 mg/kg/day (Figure 4A).

To contextualize exposure across species, murine efficacy doses (10 and 20 mg/kg/day) were translated using proportional exposure assumptions. Under this framework, 10 mg/kg/day in mice was estimated to yield approximately 167 ng·h/mL, while 20 mg/kg/day yielded approximately 333 ng·h/mL. The 30 mg/kg/day monkey dose produced exposure comparable to the lower end of the murine efficacious range. In contrast, the 90 mg/kg/day dose generated exposure exceeding that achieved at the highest murine dose.

These findings demonstrate that the primate study design encompassed both sub-equivalent and supra-equivalent exposure levels relative to murine efficacy.

Leishmanicidal efficacy in monkeys exhibited a clear exposure-dependent pattern. E_max_ modeling of the exposure–response relationship yielded an estimated EC_50_ for efficacy of approximately 209 ng·h/mL. The 30 mg/kg/day dose (∼200 ng·h/mL) approximated the midpoint of the pharmacodynamic curve, while 90 mg/kg/day (∼600 ng·h/mL) approached the plateau of maximal response (Figure 4C). These results support a classical saturable exposure–response relationship consistent with concentration-driven antiparasitic activity.

Therefore, exposure–response modeling using a sigmoidal Emax model with constrained minimum (0%) and maximum (100%) effect revealed a steep pharmacodynamic relationship (Hill coefficient = 3.04). The estimated EC_50_ was 235 ng·h/mL, corresponding to an exposure-equivalent dose of 35.7 mg/kg. EC_80_ and EC_90_ were estimated at 371 and 485 ng·h/mL, respectively (dose equivalents 56.0 and 72.8 mg/kg). The highest tested dose (90 mg/kg) exceeded the projected EC_90_ and achieved 94.5% lesion inhibition, consistent with near-maximal pharmacodynamic effect (Figure 4C).

Integration with toxicological data indicated that EC_50_ exposure remains below the NOAEL-derived exposure (∼333 ng·h/mL), suggesting a favorable therapeutic window at moderate efficacy levels. However, exposures required for the near-complete remission approach or exceed the NOAEL range, reflecting a relatively steep exposure–response profile.

Previously modeled hematologic toxicity in monkeys yielded an estimated EC_50_ of ∼1,136 ng·h/mL. Comparison of efficacy and toxicity thresholds produced a therapeutic index (EC_50_tox/EC_50_eff) of approximately 5.4. Importantly, systemic exposure at 90 mg/kg/day remained below the modeled toxicity EC_50_, suggesting partial separation between efficacious and toxicity-associated exposure ranges.

## 4 Discussion

To characterize the *in vivo* activity of 18-methoxycoronaridine (18-MC) in chronic *Leishmania amazonensis* infection, we employed a well-established BALB/c model of established leishmanial disease and quantified parasitological outcomes by limiting dilution (Le Fichoux et al., 1998). Parasite burden was quantified per whole organ following standardized homogenization, ensuring consistent biological unit comparison across experimental groups. Under these conditions, 18-MC displayed striking oral efficacy, reducing parasite burden by >99% after only 5 days of dosing both in paw and popliteal lymphonode (Table 1). This magnitude reduction is comparable to that achieved by experimental approaches that typically require longer treatment courses and/or parenteral administration, as observed when tamoxifen was administered intraperitoneally at 20 mg/kg/day for 15 consecutive days, which reduced parasite load by approximately 99% in the same host–parasite context (Miguel et al., 2008). Notably, the oral delivery and markedly shorter duration of 18-MC highlight its potential for simplified regimens, a key translational advantage in cutaneous leishmaniasis where adherence and tolerability remain critical barriers.

We next explored whether extending exposure could promote sterile cure. Increasing the oral regimen to 10 days did not eradicate the infection, although it maintained consistently high parasite reductions above 99% (Table 1). This pattern is consistent with chronic *L. amazonensis* infection in susceptible BALB/c mice, in which lesion regression and marked suppression of parasite burden do not necessarily translate into sterile cure, indicating persistence of residual parasites despite clinical improvement (Present et al., 2024). Similarly, sterile cure has been difficult to achieve even with prolonged dosing schedules for other candidates. In the case of LQB-118 (a naphthopterocarpanquinone), significant control of lesion growth and parasite load required extended, intermittent administration (4.5 mg/kg/day, 5 days per week) over 85–105 days, yet complete parasitological clearance was not reported (da Cunha-Júnior et al., 2011). Collectively, these observations suggest that, in highly susceptible hosts, shortening parasite burden to near-sterility may be feasible pharmacologically, but absolute clearance may require either longer exposure, optimized combinations, or host-directed strategies that promote durable immune-mediated elimination.

In non-human primates, oral 18-MC produced a clear dose-response relationship with meaningful clinical benefit. In *Chlorocebus aethiops*, 18-MC at 30 or 90 mg/kg/day for 28 days reduced lesion burden by 67% and 98%, respectively, accompanied by robust re-epithelialization and improvement of satellite lesions, without overt clinical or behavioral abnormalities during treatment (Figures 2, 3). These outcomes compare favorably with immunotherapy-adjunct strategies in primate cutaneous leishmaniasis models. CpG ODN D35 improved lesion outcomes when combined with an abbreviated low-dose pentavalent antimonial course in macaques infected with *L. major*, accelerating re-epithelialization without evident toxicity, even at doses above those required for efficacy (Thacker et al., 2020). Unlike D35, which acts primarily via immune activation in combination with antimonials, 18-MC achieved marked lesion reduction as a standalone oral agent, supporting intrinsic antileishmanial potency in primates and motivating future evaluation in rational combinations (Delorenzi et al., 2002).

Across species, the concordance between murine and primate outcomes supports a consistent pharmacodynamic signal for 18-MC. In mice, oral dosing at 10–20 mg/kg/day produced >99% reductions in parasite load in established infection, while in non-human primates 90 mg/kg/day approached near-complete lesion control with favorable tolerability. This cross-species alignment is notable given the recognized low efficiency and high failure rate in antileishmanial drug discovery, in part driven by limitations of surrogate preclinical models and the resulting translation gap (Olías-Molero et al., 2021; Lafleur et al., 2024). Moreover, antileishmanial efficacy work overwhelmingly relies on rodent models, whereas non-human primate models are less frequently employed despite their translational value (van der Ende and Schallig, 2023; André et al., 2020). Accordingly, non-human primate efficacy datasets for chemotherapeutic small molecules in cutaneous leishmaniasis remain comparatively limited, and studies spanning such higher-order models provide an important bridge toward clinical translation (van der Ende and Schallig, 2023).

Because First Time-in-Human (FTiH) progression requires a strong safety rationale, we conducted acute and repeated-dose toxicology in line with internationally accepted frameworks. Contemporary OECD approaches emphasize humane endpoints and tiered designs that reduce reliance on classical LD_50_​ testing (Botham, 2004), and acute oral toxicity methods are formalized in Test Guidelines 420 (fixed dose), 423 (acute toxic class), and 425 (up-and-down) (OECD, 2001a; OECD, 2001b; OECD, 2022). Although females are frequently selected as the default sex for acute testing based on historical sensitivity patterns (Lipnick et al., 1995; OECD, 2022), our data revealed pronounced sex-dependent susceptibility, with males exhibiting lower tolerance thresholds than females (Table 2). Specifically, the estimated LD_50_​ in males (130 mg/kg) was substantially lower than in females (≥300 mg/kg), and male deaths occurred rapidly (within the first hours post-dose), whereas females showed delayed and less frequent lethality at higher doses. This sex divergence underscores the value of evaluating both sexes during early safety profiling when a compound’s pharmacology plausibly intersects neuroendocrine or neuromodulatory pathways.

Mechanistically, the higher susceptibility in males may reflect sex-dependent differences in neuroactive signaling systems and/or pharmacokinetics. 18-MC and related iboga alkaloids have been reported to interact with opioid-linked signaling pathways, including µ-opioid receptor-coupled G-protein activation (Pearl et al., 1997; Antonio et al., 2013). In parallel, substantial sex differences are recognized in κ-opioid pharmacology and broader opioid responses in both humans and animal models (Rasakham and Liu-Chen, 2011). While these data do not establish causality, they provide a plausible biological context for differential neurobehavioral and systemic responses after high-dose exposure. Additionally, sex-dependent metabolic or distribution differences may contribute; notably, sex differences have also been reported in metabolic phenotypes and in the physiological response to 18-MC in obesity-related experiments (Taraschenko et al., 2011).

Histopathological assessment (Supplementary Material) further supported a favorable therapeutic window at efficacious doses, while identifying target-organ liabilities at supratherapeutic exposure. No relevant microscopic alterations were observed in major organs at 50–100 mg/kg in acute settings (data not shown), whereas kidney and liver changes emerged at ≥200 mg/kg, including glomerular alterations consistent with impaired filtration and hepatic nuclear/chromatin changes suggestive of cellular stress. In repeated-dose settings, kidney findings were detectable at 50 mg/kg and became more prominent at 100 mg/kg, indicating that renal tissue may represent a primary target organ under sustained exposure. Such patterns are consistent with established concepts in systemic toxicology where delayed organ injury can arise from cumulative exposure, adaptive stress responses, and/or metabolic activation (OECD, 2008). More broadly, hepatic injury risk in small-molecule development is often linked to bioactivation and reactive intermediates generated by drug-metabolizing enzymes, including cytochrome P450 systems (Guengerich, 2006; Chen et al., 2015), while drug-induced nephrotoxicity can reflect multiple convergent mechanisms, including hemodynamic changes and tubular/interstitial injury (Kwiatkowska et al., 2021). Importantly, the overall profile of 18-MC in mice remained compatible with continued development: clinical, biochemical, and histopathological readouts supported a NOAEL of 50 mg/kg/day for 30 day repeated dosing, with adverse findings confined to higher multiples of the effective dose (Table 4 and 5).

NHP safety studies reinforced these conclusions. In *Cynomolgus monkeys*, a 14 day oral gavage study at 50, 150, and 300 mg/kg/day with a 4 week recovery phase indicated that 18-MC was generally well tolerated, without mortality during dosing or recovery (technical report, unpublished). At the highest dose, occasional emesis under fasted conditions was observed, and minimal hepatic vacuolation and mild renal tubular changes were reported, with resolution after the recovery period, consistent with non-progressive and reversible adaptive responses. Together with the murine findings, these results support a wide safety margin and provide a robust basis for selecting conservative starting doses in early clinical evaluation.

The present study provides quantitative evidence that 18-MC exerts exposure-dependent leishmanicidal activity in non-human primates. The observed E_max-type_ relationship between systemic exposure (AUC) and antiparasitic efficacy strongly supports a concentration-driven pharmacodynamic mechanism. The estimated effective EC_50_ (∼209 ng·h/mL) indicates that relatively moderate systemic exposure is sufficient to achieve significant parasite reduction. Importantly, the 90 mg/kg/day dose in NHP achieved approximately threefold higher exposure than the modeled efficacy EC_50_, placing it within the near-maximal response region of the pharmacodynamic curve. This finding suggests that suboptimal exposure—rather than intrinsic pharmacologic inefficacy—may limit antiparasitic activity at lower doses.

The exposure-response profile observed for 18-MC demonstrates a steep sigmoidal relationship (Hill coefficient ≈3), indicating that incremental increases in systemic exposure near the EC_50_ translate into substantial gains in lesion inhibition. This pharmacodynamic behavior contrasts with the relatively shallow exposure-response relationships described for miltefosine in both preclinical and clinical leishmaniasis models, where Hill coefficients are typically <2 and efficacy is strongly influenced by prolonged exposure and cumulative drug accumulation (Dorlo et al., 2012; Mbui et al., 2019; Wasunna et al., 2016). While miltefosine and amphotericin B exhibit high intrinsic potency, they are frequently constrained by narrow therapeutic margins or dose-limiting toxicities. In contrast, the steep and predictable exposure–efficacy relationship observed here suggests a more direct pharmacodynamic mechanism within the therapeutic range (Sundar and Chakravarty, 2010).

Notably, systemic exposure at 90 mg/kg/day (∼600 ng·h/mL) remained substantially below the estimated EC_50_ for hematologic toxicity (∼1,136 ng·h/mL). This separation resulted in an estimated therapeutic index of approximately 5.4, suggesting partial but meaningful dissociation between efficacy and toxicity exposure thresholds. Demonstrating this separation in a primate model significantly strengthens translational confidence and supports exposure-based dose optimization strategies. Comparison with murine efficacy data demonstrated that 30 mg/kg/day in monkeys generated exposure comparable to lower murine effective doses, while 90 mg/kg/day exceeded the exposure achieved at the highest murine dose. Such exposure-normalized translation is critical for rational progression toward human dose selection.

Despite the strength of the translational modeling framework, several limitations must be acknowledged: First, exposure estimates in this analysis were derived under an assumption of linear pharmacokinetics within the evaluated dose range and while prior toxicokinetic data support a linearity, formal nonlinear mixed-effects modeling was not conducted, so inter-individual variability and parameter uncertainty were not fully characterized. Second, efficacy modeling relied on limited exposure–response data points, and parameter estimation used deterministic grid-search optimization rather than nonlinear regression with formal confidence intervals. As a result, EC_50_ and Emax estimates should be considered exploratory rather than definitive pharmacometrics parameters. Third, systemic exposure (AUC) served as a surrogate for pharmacologically active concentrations at the infection site. Tissue distribution and intracellular drug concentrations, particularly within macrophages, were not directly quantified, so mechanistic interpretation of exposure–effect relationships remain indirect. Finally, toxicity and efficacy modeling were conducted independently, and a fully integrated pharmacokinetic–pharmacodynamic (PK–PD) safety model incorporating time-dependent hematologic recovery was beyond the scope of this analysis.

In spite of these limitations, the integrated exposure–response analysis demonstrates a quantifiable therapeutic window in a primate model and supports the translational viability of 18-MC as a candidate antileishmanial agent. The data suggest that exposure, rather than administered dose alone, should guide further development decisions. Collectively, these findings provide a quantitative foundation for exposure-guided dose optimization in future translational or early clinical investigations.

Finally, these datasets on efficacy and safety enable translational planning for FTiH studies. Using standard FDA allometric scaling principles to derive human equivalent doses from animal NOAELs and pharmacologically active exposures, an HED of approximately 16 mg/kg/day is supported as a plausible regimen for Phase I evaluation, subject to protocol-specific safety factors and clinical monitoring (FDA, 2005; Nair and Jacob, 2016). In sum, 18-MC combines potent antileishmanial activity in chronic murine infection with dose-responsive clinical benefit in non-human primates and a safety profile compatible with advancement (Sundar and Chakravarty, 2013). Future experiments should be conducted to evaluate the 18-MC mechanism of action and its pharmacokinetics in NHP, in preparation for the FTiH clinical protocol.

## 5 Conclusion

This study presents robust pre-clinical evidence supporting 18-methoxycoronaridine (18-MC) as a promising oral therapeutic candidate for cutaneous leishmaniasis caused by *Leishmania (Leishmania) amazonensis*. In the chronic BALB/c infection model, short-course oral administration achieved a rapid and consistent reduction in tissue parasite burden exceeding 99% within 5 days, demonstrating higher efficacy than parenteral Glucantime the standard control, and for other reported experimental agents. Extending treatment to 10 days sustained this suppression, although a sterile cure was not achieved. This outcome aligns with the persistent nature of chronic *L. amazonensis* infection in susceptible hosts and reinforces the need to optimize exposure–response relationships and investigate rational combination therapies to achieve durable parasite clearance.

Efficacy translated to a non-human primate model (*Chlorocebus aethiops*), where oral administration 18-MC produced clear dose-dependent clinical benefit. Treatment for 28 days reduced lesion burden by 67% at 30 mg/kg/day and by 98% at 90 mg/kg/day, with marked re-epithelialization and improvement of satellite lesions, without any clinical or behavioral toxicity. The concordance between rodent parasitological outcomes and primate clinical responses strengthens confidence in the pharmacodynamic consistency of 18-MC across species and highlights the feasibility of scalable oral regimens.

Safety profiling provides additional support for further development. In mice, 18-MC demonstrated no toxicity at therapeutically relevant doses, with adverse effects and target-organ microlesions (primarily renal and hepatic) observed only at high exposures. A conservative analysis established the no-observed-adverse-effect level (NOAEL) for repeated dosing at 50 mg/kg/day in both sexes, indicating a substantial safety margin relative to efficacious doses. Complementary studies in cynomolgus monkeys (*Macaca fascicularis*) indicated good tolerability up to 300 mg/kg/day, with only mild, reversible findings at the highest exposures and full recovery after cessation, reinforcing the reversibility of observed changes and the overall safety margin.

Taken together, these results identify 18-MC as an orally bioavailable antileishmanial drug with potent activity, robust rodent–primate translational concordance, and a safety profile suitable for first-in-human evaluation. The combined efficacy and toxicology/PK evidence supports progression to formal GLP-enabling studies and Phase I clinical development, with an estimated human equivalent dose of approximately 16 mg/kg/day as a rational starting point for dose-escalation under careful clinical monitoring. Future work should focus on refining dosing strategies to maximize the probability of parasitological clearance, evaluating the 18-MC mechanism of action and its pharmacokinetics in NHP, and preparing for the FTiH clinical protocol.

## Data Availability

The original contributions presented in the study are included in the article/Supplementary Material, further inquiries can be directed to the corresponding author.
