# Supplementary material for: Oral 18-methoxycoronaridine activity in simian and murine Leishmania amazonensis infection
Source: Front Pharmacol. 2026 Apr 10;17:1780255. doi: 10.3389/fphar.2026.1780255 (PMC13106002; doi:10.3389/fphar.2026.1780255)
Supplement: Supplementary file 1 [file Supplementaryfile1.pdf]

SUPPLEMANTARY MATERIALS

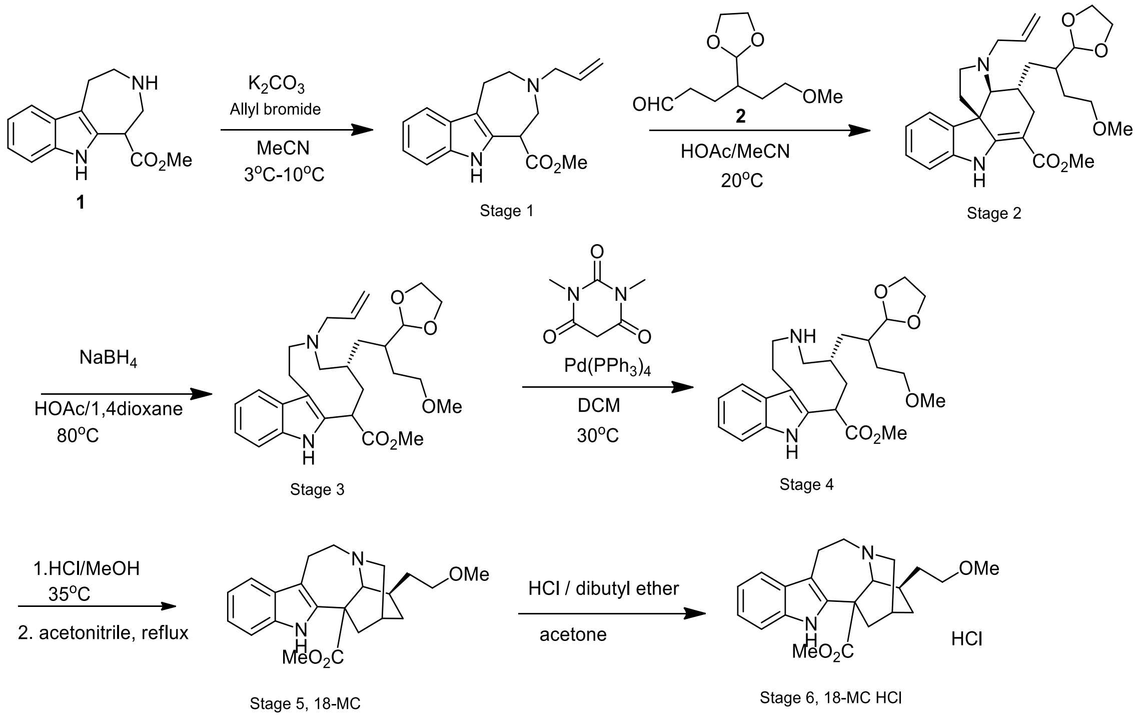

Figure S1 – Manufacturing schematic description of 18-MC. The detailed procedure is not disclosed due to patent and industrial protection.

Elucidation of Structure and other Characteristics

The molecular structure of 18-Methoxycoronaridine hydrochloride has been determined on behalf of Savant HWP by Professor John Blacker, University of Leeds, Institute of Process Research and Development (iPRD).

To confirm the structural identity of 18-MC HCl. The following tests were performed:

- Crystal x-ray analysis
- ^{1}H NMR (500MHz) in $CDCl_3$
- ^{13}C NMR (500MHz) in $CDCl_3$

The calculated XRD powder diffraction of the recrystallised 44A is shown in Figure 1.1-1. The single-crystal x-ray study confirms the structure of 18-MC HCl (Figure 1.1-2) and is similar to that reported by Kuehne (Kuehne and Waterman, 2012).

Samples were examined using ^{1}H and ^{13}C NMR (500MHz) in $CDCl_3$. The observed chemical shifts and related data in all three samples closely matched those reported by Banderage et al. (1999). Currently, no reported correlation has been found between the 18-MC HCl structure and NMR data. Several 2d NMR experiments (COSY, HMQC and HMBD) were performed to correlate ^{1}H and ^{13}C NMR signals with the structure, Figure 1.1-3, and the results are reported in Table 1.1-1. The NMR correlations confirm the identity of the structure, with the chemical shift that is associated with each of the carbons in the molecule. The analysis of the structure shows there are four chiral centers. Of the four chiral centers three of them are locked during the manufacturing process [C14(S*),

C16(R*), C21 (R*)] and the one chiral center is located at C20 (S*), resulting in a racemic mixture of two enantiomers in the synthesized API.

In conclusion, the structure of 18-MC HCl produced by Aesica Pharmaceutical LTD is confirmed to be the same structure as that reported by Keuhne.

Figure S2.1-1: XRD powder pattern of 18-MCHCl using Mercury

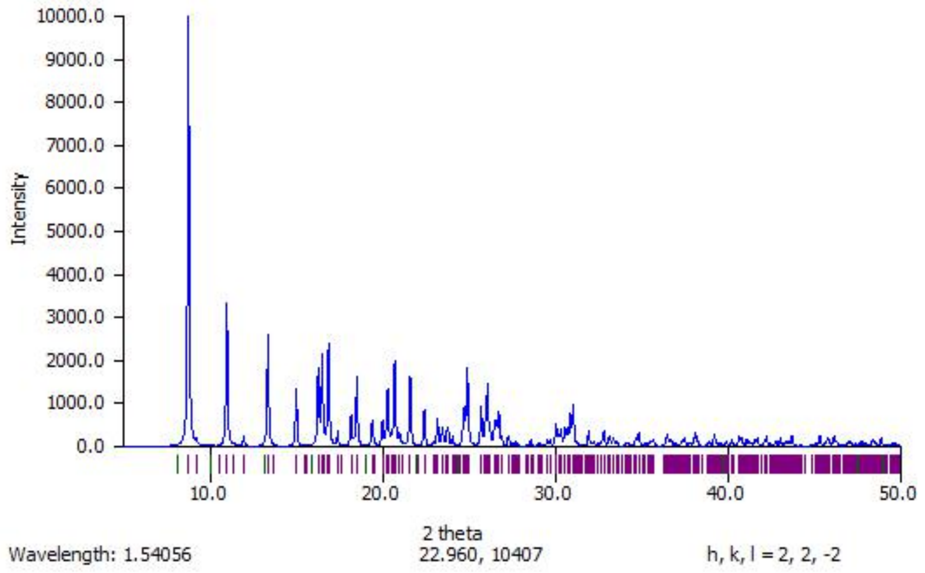

Figure S2.1-2: Single Crystal x-ray structure of 18-MC HCl sample 44-2

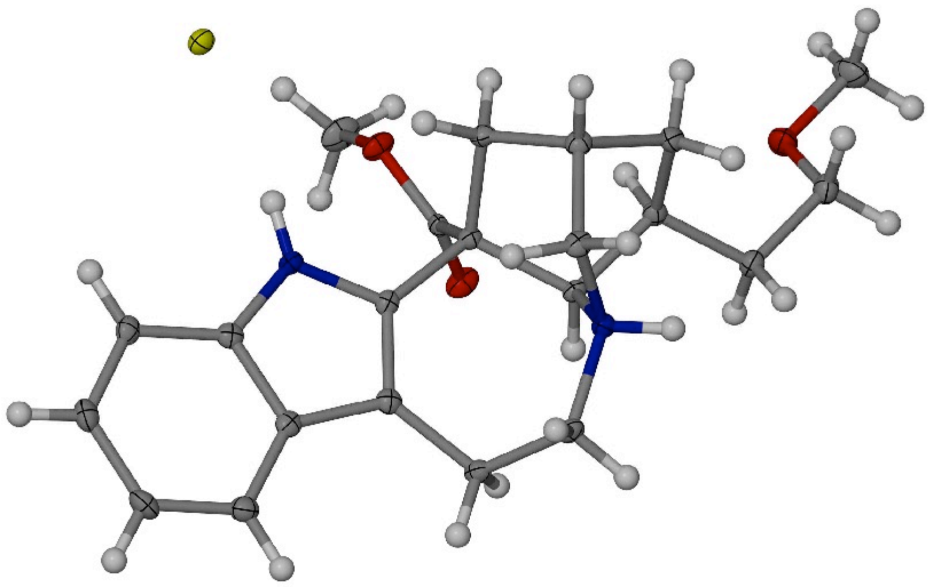

Figure S2.1-3: ^{1}H and ^{13}C Assignments based on COSY, DEPT, HMQC and HMBD NMR data

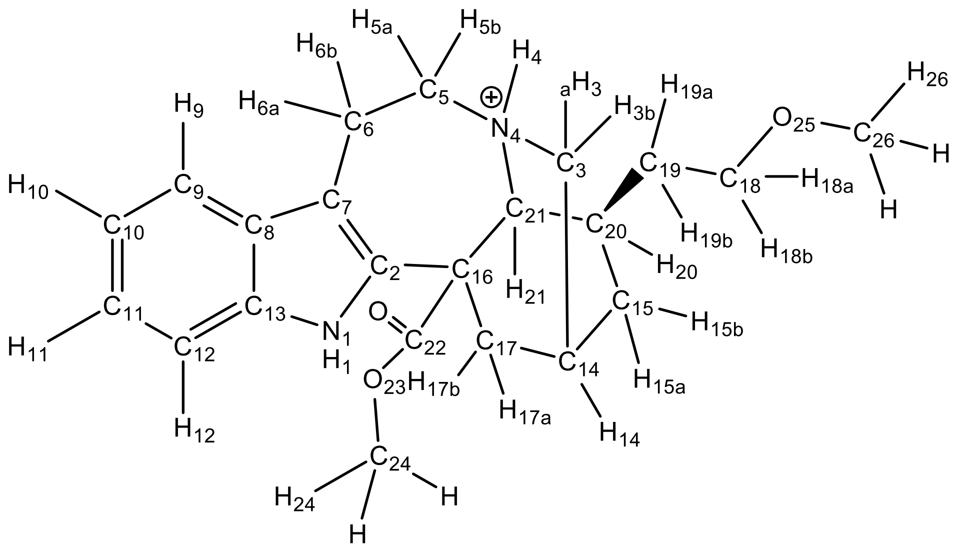

Table S1.1-1: ^{1}H and ^{13}C structural assignments based on COSY, DEPT, HMQC, and HMBD NMR data

Atom	Chemical Shift (ppm)	Atom	Chemical Shift (ppm)	Atom	Chemical Shift (ppm)
**N1**		H1	8.07, brs, 1H		
**C2**	132.77				
**C3**	53.19	**H3a**	3.94, m, 1H	H3b	2.92, dd, 5.2Hz, 2.8Hz
**N4**		**H4**	11.5, b, 0.7H		
**C5**	55.32	**H5a**	4.08, m, 1H	H5b	3.60, m, 1H
**C6**	18.88	**H6a**	3.40, m, 1H	H6b	3.17, dt, 6.8Hz, 2.4Hz, 1H
**C7**	108.53				
**C8**	127.14				
**C9**	118.45	**H9**	7.49, d, 3.2Hz, 1H		
**C10**	120.48	**H10**	7.16, t, 4.0Hz, 1H		
**C11**	123.63	**H11**	7.19, t, 4.0Hz, 1H		
**C12**	111.04	**H12**	7.32, d, 3.2Hz, 1H		
**C13**	135.77				
**C14**	25.20	**H14**	2.23, bm, 1H		
**C15**	28.06	**H15a**	1.99, bm, 1H	H15b	2.00, bm, 1H
**C16**	53.19				
**C17**	35.63	**H17a**	2.67, dd, 4.4Hz, 0.1Hz, 1H	H17b	2.24, dd, 4.4Hz, 0.1Hz, 1H
**C18**	69.84	**H18a**	3.50, m, 1H	H18b	3.45, m, 1H
**C19**	31.55	**H19a**	2.57, m, 1H	H19b	2.23, bm, 1H
**C20**	32.88	**H20**	2.01, m, 1H		
**C21**	58.51	**H21**	4.46, b, 1H		
**C22**	172.33				
**O23**					
**C24**	53.72	**H24**	3.78, s, 3H		
**O25**					
**C26**	58.58	**H26**	3.33, s, 3H		

Analytical Procedures

The analytical methods used for the analysis and release testing of 18-MC HCl are described below.

<u>Identification: IR</u>
An infrared spectrum over the range 4000 to 450 cm^{-1}.

<u>Identification: Chloride</u>
A sample of 18-MC HCl is dissolved in water, acidified, and a 0.1M Silver Nitrate solution is added. A curdled white precipitate is formed. The precipitate is separated from the solution and washed. Suspend the precipitate in 2ml of water and add 1.5ml 10M Ammonia solution.
A positive result is indicated by the precipitate dissolving readily in the solution, except for a few large particles, which dissolve slowly.

<u>Chloride Assay (on dried basis)</u>
Dissolve a sample of 18-MC HCl in Methanol and 2M Nitric acid. Add water and swirl gently to mix. Titrate potentiometrically with 0.1M Silver nitrate solution, and record the endpoint potentiometrically.

<u>Assay/Purity & Related Substances by HPLC</u>
The assay, related substances, and purity of 18-MC and 18-MC HCl are determined by HPLC analysis. For the assay, a known standard is used to calculate the amount of 18-MC HCl in the sample. A summary of the method is below.

<u>Instrument parameters</u>

Column:	Phenomenex Gemini C18 3µm 150 x 4.6mm
Flow Rate:	1.5ml/min
Temperature:	25°C
Injection Volume	10µl
Detector:	284nm
Run Time	60 minutes
Isocratic Condition	67% mobile phase A, 33% mobile phase B
Retention time	10 minutes +/- 1 minute
Run time	60 minutes

<u>Residual Solvents</u>
Residual solvents are determined by gas chromatography head space analysis. A summary of the method is below.

<u>Instrument parameters</u>

Column:	DB-624 60m x 0.32mm x 1.8mm film thickness
Detector:	Flame ionisation
Oven temperature:	40ºC (hold for 1 min)<br>then 10 ºC / min to 170ºC (hold for 3.0min)<br>then 20 ºC / min to 260 ºC (hold for 2.5min)
Injection temperature:	260ºC.
Detector temperature:	280ºC.

Split ratio: 5:1
Column head pressure: 10 psi, constant pressure
Run time: 24 minutes
Carrier gas: Nitrogen

Acetic acid by UHPLC

Residual acetic acid is determined by UHPLC. A summary of the method is below.

Instrument parameters

Instrument: Acquity H Class UHPLC or equivalent
Column: Acquity T3 50mm x 2.1mm, 1.7µm particle size
Flow Rate: 0.5ml/min
Temperature: 30°C
Injection Volume: 10µl
Detector: 210nm
Run Time: 3 minutes
Gradient Program:

Time (mins)	%B
0	0
1	0
1.1	100
2	100
2.1	0
3	0

Retention time; 0.52minutes +/- 0.05 minute

Enantiomeric Analysis

18-MC HCl is a racemic mixture of two enantiomers. The analysis for chiral consistency is an HPLC method using a chiral column.

Instrument parameters

Column: Daicel Chiralpak AD-H 5µm 250 x 4.6mm
Flow Rate: 1ml/min
Temperature: 25°C
Injection Volume 10µl
Detector: 285nm
Run Time 18 minutes
Retention time R1 8 minutes +/- 1 minute, R2 11 minutes +/- 1 minute

Figure 1. Representative Chromatogram of a 10.0 µg 18-MC HCl/mL Calibration Standard

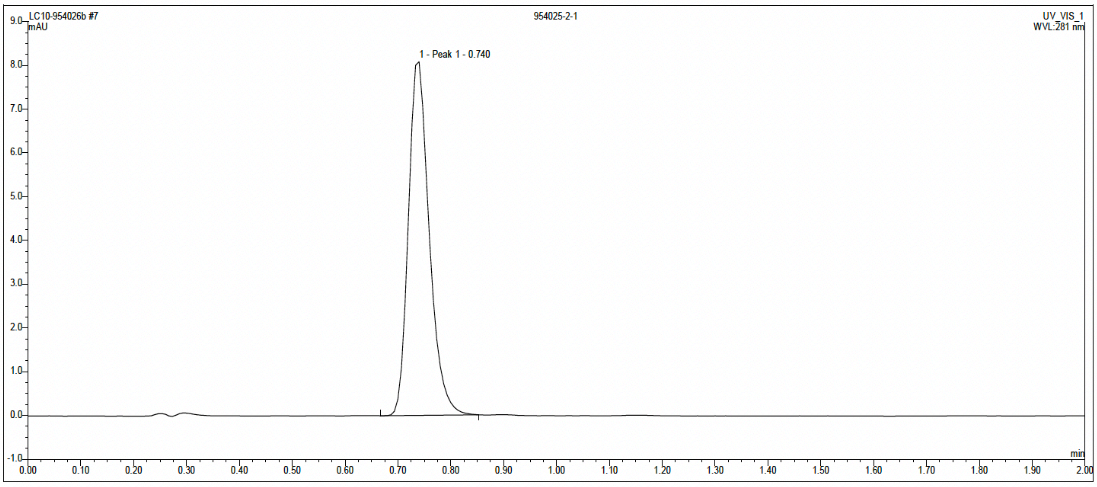

Figure 2. Representative Chromatogram of a Processed 10.0 mg 18-MC HCl/mL QC Sample

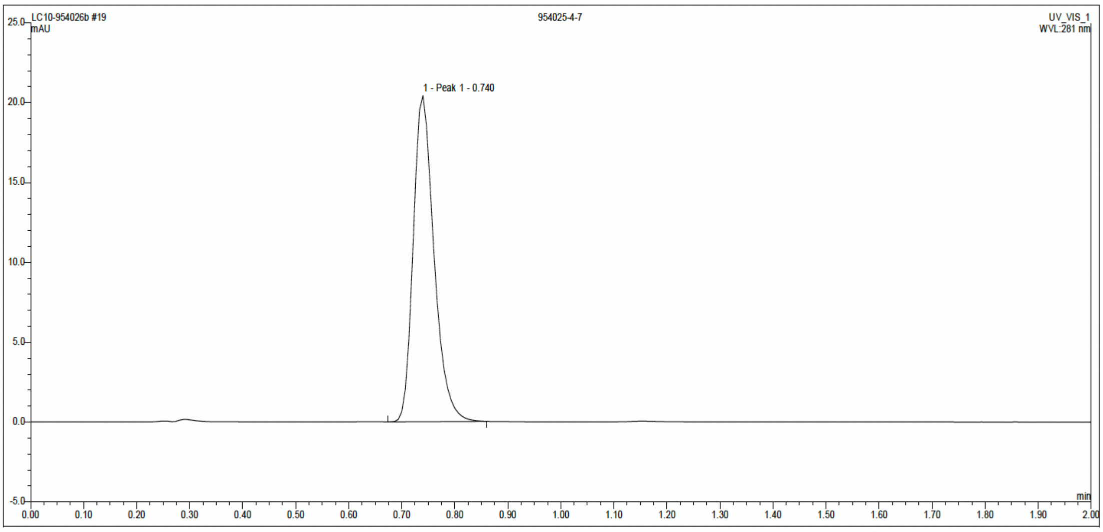

Figure 3. Representative Chromatogram of a Processed 40 mg 18-MC HCl/mL Formulation Sample

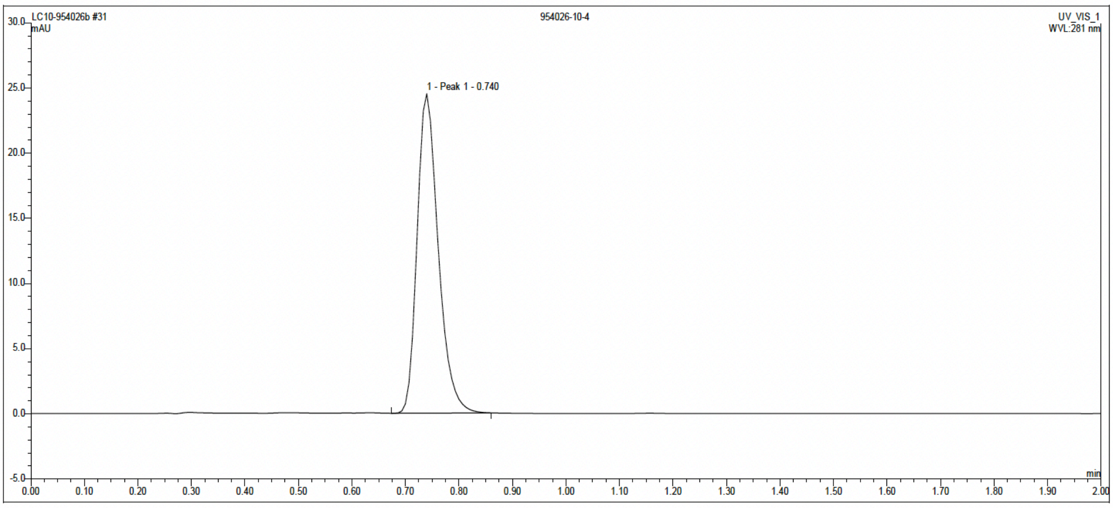

Figure 4. Representative Chromatogram of a Processed Control Group Formulation Sample

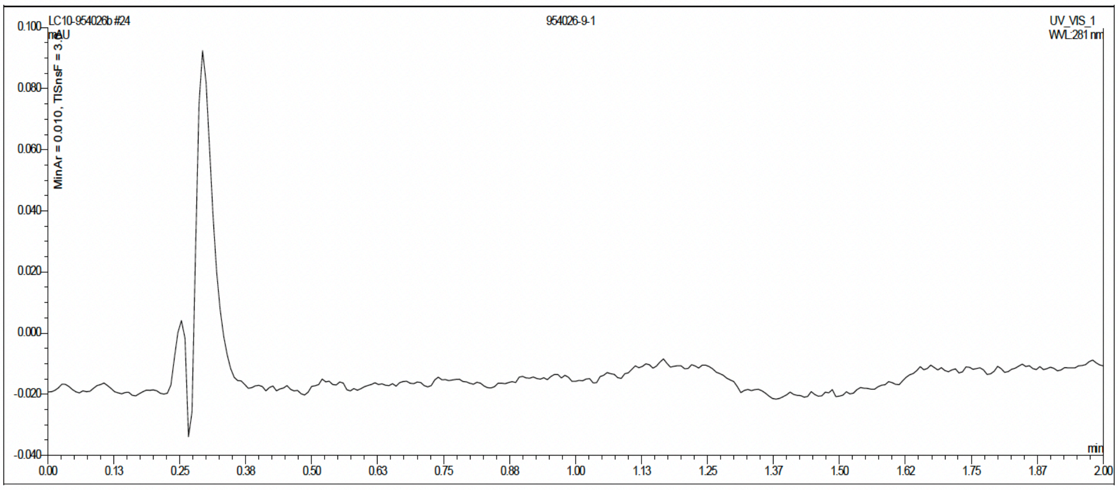

Histopathological Analysis of 18-MC Toxicity

1. Acute Toxicity Morphological Analysis

Histological examinations of the **spleen and the brain** of animals that progressed to death following acute treatment with 200 mg/kg of 18-Methoxycoronaridine (18-MC) revealed no significant morphological alterations. These results were comparable to the control groups evaluated in the same experimental sets.

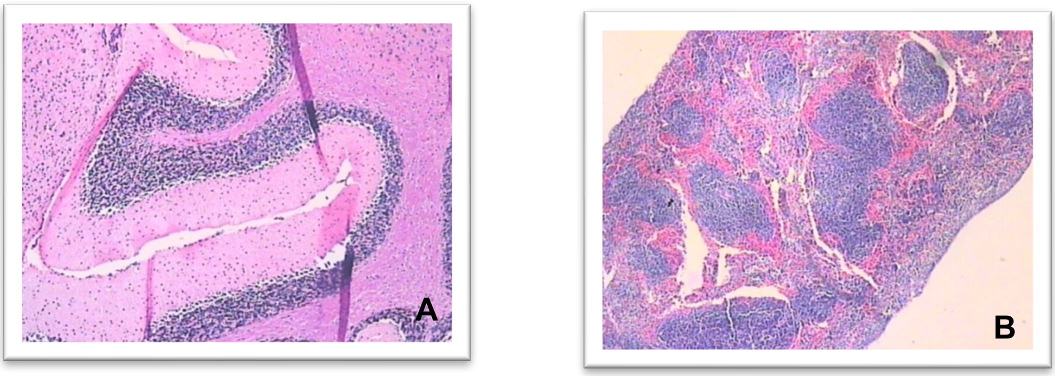

Figure S3. Histological analysis of the kidney (A) and Spleen (B) from animals treated with 200 mg/kg of 18-MC. No morphological changes were evident even at toxic doses. Staining: Hematoxylin-Eosin (H&E). Magnification: (A) 100X; (B) 50X.

In contrast, histopathological analysis of the **kidneys and the liver** of animals treated with single doses of 200 or 300 mg/kg of 18-MC revealed tissue damage that could be considered co-responsible for the drug-induced mortality observed in these subjects.

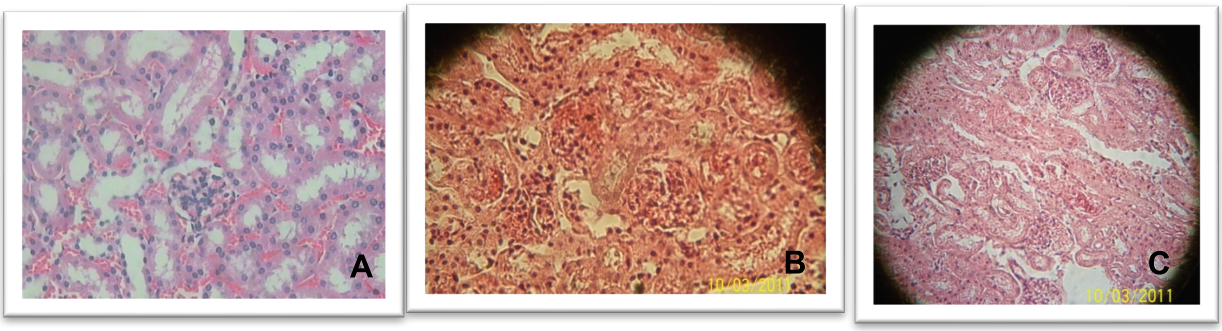

Figure S4 – Histological sections of kidney from CTRL animals (A) and treated with 200 mg/kg (B) and 300 mg/kg (C) of 18-MC. Collapse of Bowman's capsules can be observed in the treated animals, which could justify some of the toxic effects observed at these concentrations. Staining: Hematoxylin-Eosin. Magnification: 200X.

- **Renal Impact:** Treated animals exhibited collapse of the Bowman's capsules, which could justify the toxic effects observed at these concentrations.
- **Hepatic Impact:** Observations suggested increased nuclear size, potential chromatin condensation, and cellular retraction within the hepatic parenchyma.

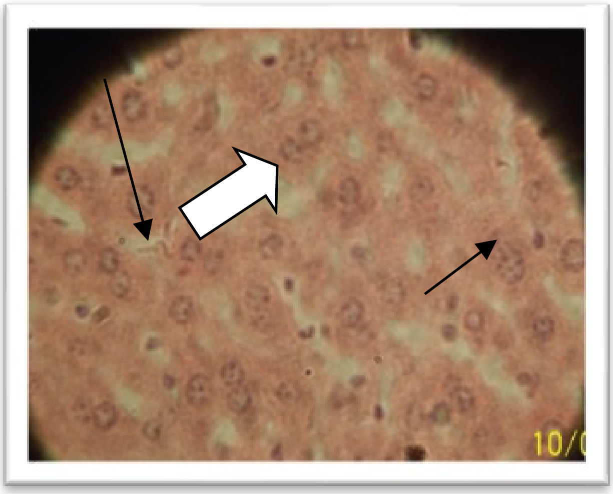

Figure S5. Histological section of the liver from an animal treated with 200 mg/kg of 18-MC. Fine arrows suggest nuclear enlargement and potential chromatin condensation; the wide arrow indicates possible cellular retraction. Staining: H&E; Magnification: 200X.

2. Repeated-Dose (Chronic) Toxicity

The histopathological findings from the 5-day repeated-dose study closely mirror the data obtained in the acute toxicity trials.

- **Preserved Organs:** The spleen, brain, heart, and lungs maintained well-preserved tissue architecture across all studied concentrations, remaining comparable to control groups.
- **Target Organs:** The kidneys and liver appear to be the primary targets for potential tissue damage caused by 18-MC. Significant alterations were specifically noted at doses of 50 and 100 mg/kg/day.

2.1. Organ Architecture Preservation (Brain and Heart)

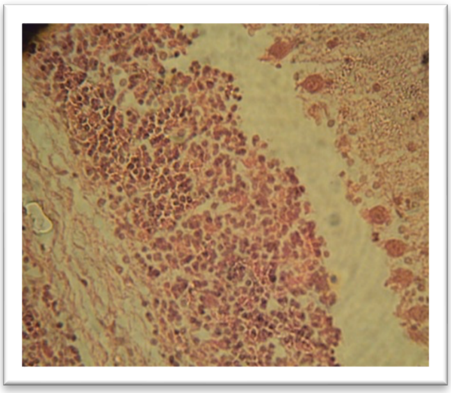

Figure S6. Brain of an animal treated with a daily dose of 20 mg/kg of 18-MC for five days, showing good preservation of tissue architecture. Staining: H&E; Magnification: 400X.

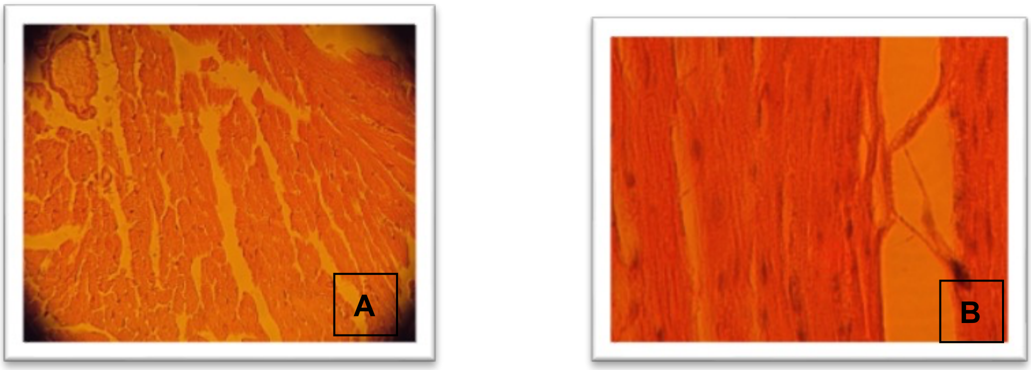

Figure S7. (A) Heart of an animal treated with 20 mg/kg of 18-MC for five days, showing preserved tissue architecture. (B) Histological section of the heart from untreated animals (CTRL). Staining: H&E; Magnification: 400X.

2.2. Renal Susceptibility and Glomerular Impact

The kidney was found to be the organ most susceptible to 18-MC in both acute and repeated-dose studies. The collapse of Bowman's capsules may compromise glomerular filtration and lead to the accumulation of fluids and toxic substances, which may be implicated in the toxicity observed at higher doses leading to death.

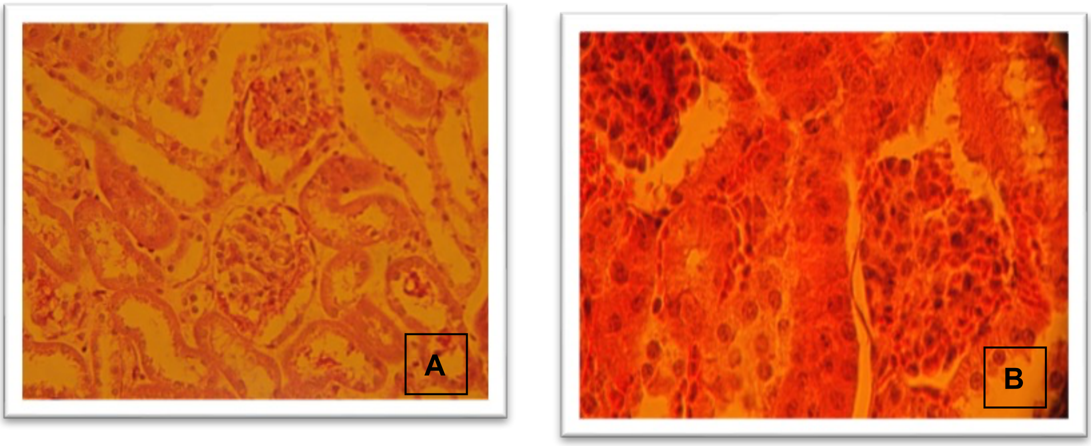

Figure S8. (A) Kidney of an animal treated with 20 mg/kg of 18-MC for five days, presenting preserved tissue architecture and glomerular structures, without retraction of the Bowman's capsule. (B) Histological section of the brain (CTRL). Staining: H&E; Magnification: 400X.

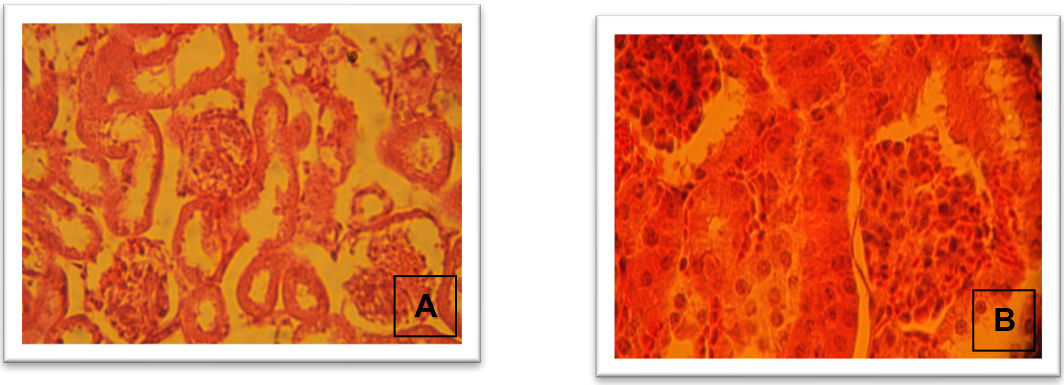

Figure S6 – **A** - Kidney of an animal treated with a single daily dose for five days of 50 mg/kg of 18-MC showing collapse of some glomeruli with reduction of Bowman's space. **B** – Histological section of the kidney of untreated animals (CTRL). Staining: Hematoxylin-Eosin. Magnification: 400X.
